# A Theory of Mine: My Perspective on Perspective-Taking

**DOI:** 10.1089/aut.2022.0002

**Published:** 2022-12-13

**Authors:** Gyasi Burks-Abbott

**Affiliations:** ^1^ LEND Program, Boston Children's Hospital, Boston, Massachusetts, USA.; ^2^ The Institute for Community Inclusion, University of Massachusetts Boston, Boston, Massachusetts, USA.

**Keywords:** self-advocate perspectives, Black/African American autistics, theory of mind, double empathy

Though I was born both Black and autistic, the latter identity was not confirmed until I was 17 years old. Before that, my problems were attributed to many different causes including emotional disturbance, a learning disability, lack of intelligence, or just plain laziness. Sometimes my difficulties were linked to my race. When I failed the math portion of a science examination, my fifth grade teacher wrote what was then the slogan of the United Negro College Fund across the top of my paper: “A Mind is a Terrible Thing to Waste.”

My fifth grade teacher need not have worried. Being raised by an academic who instilled in me a love of learning from a young age, my mind was going to be just fine. In addition to helping me with my homework and advocating for me with my teachers, my mother also made education an organic part of my life outside of school. A magnetic alphabet set on the refrigerator and labels placed around the house immersed me in language, as did the hours of conversation my mother and I would have about everything from current events to interpersonal relationships. We also watched Jeopardy, the popular television quiz show, every night.

My mother also managed to make blackness an integral part of my education, even though I went to predominately White schools that offered mostly Euro-centric curricula. I was always enrolled in our local public library's summer reading program, and my mother would have me read books by and about African Americans. My mother's field was English with a specialization in African American literature and film, and she would discuss aspects of her work with me—particularly how Hollywood cinema shaped the popular perception of African Americans. She also took me to conferences where I would get to meet other Black scholars.

Shortly after my diagnosis, my mother introduced me to the work of Henry Louis Gates, Jr., a Black literary scholar (and current host of the PBS television series “Finding Your Roots”) who documented how Enlightenment philosophers disparaged the humanity of Blacks and defended the institution of slavery. According to Gates, the slave narrative was part of the Black response to this blanket condemnation by the 18th century intellectual establishment. As he asserts in *Figures in Black: Words, Signs, and the “Racial” Self*^1^:

I hope that it is obvious that the creation of formal literature was no mean matter in the life of the slave, since the sheer literacy of writing was the very commodity that separated animal from human being, slave from citizen, object from subject. Reading, and especially writing, in the life of the slave represented a process larger than even “mere” physical manumission, since mastery of the arts and letters was Enlightenment Europe's sign of that solid line between human being and thing (24–25).

As an African American and as an autistic, it was, perhaps, inevitable that I would see parallels between the two conditions—particularly the link between the slave narrative and the autistic narrative. The assertions some experts made about autistics were, for me, reminiscent of the claims made about Blacks during the Enlightenment. Both groups were deemed incapable of higher thought and complex emotion, and both groups fought back by telling their own stories.

A turning point in my story occurred three decades ago when, after years of missed opportunities and misunderstandings, I was finally diagnosed with infantile autism. As much as the new label accurately captured my life experience, some of the experts' pronouncements about autism really bothered me. As a very affectionate person, I could not stand the idea that autistics cannot stand to be touched,^2^ and I felt personally insulted by the notion that autistics were literal thinkers lacking in empathy.^3^ My mother suggested that since I had chosen psychology as one of my college majors (the other being English), I was in a perfect position to confront my problems with autism discourse head on.

For an independent project, I conducted a study to test the validity of the diagnostic criteria for autism. I distributed a survey to two sets of people—autistics and people who work with autistics—asking the former to what extent they agreed with statements such as “I cannot stand to be touched” and the latter to what extent they thought the autistics they worked with would agree with such statements.

Short of the definitive refutation I had hoped for of what I considered autism stereotypes, I discovered a spectrum of responses from both my autistic and nonautistic informants. Some autistic informants, for instance, did express a disdain for physical contact, circling “no” in response to my question about whether or not they liked to be touched. But, as a group, they were not monolithic. There were a range of responses that reflected the diversity and complexity of the condition.

And, what I found particularly heartening, my nonautistic informants did not answer the questions based on some abstract concept of autism but instead on the individual characteristics of the autistic informants they knew personally, something they made clear in the marginalia and comments section of the survey. I decided that there was indeed an autism stereotype, but that like most stereotypes it came from a ripple of reality blown out of proportion into a tidal wave of generalizations.

I also realized part of my objection to autistics as literal thinkers stemmed from a value judgment that placed abstract thought and language on a pedestal. However, the literal and the concrete are just as significant. Imagine this scenario: A driver gets pulled over by the police, and the officer says “sir, you just ran that stop sign; did you not see it?” The driver responds, “of course I saw the stop sign, officer, but I didn't take it literally.” In terms of social behavior, the driver's response would be just as inappropriate as that of the autistic dinner guest who, in a scenario often cited by experts, simply says “yes” when asked whether he can pass the salt. Whereas one was being literal, and the other was not being literal, both failed to respond appropriately to the situation. Context is everything.

As for the autistic dinner guest who made the faux pas of answering the question asked instead of responding to the meaning of what was implied, there was an episode of the 1990s political drama “The West Wing” in which responding to the implied meaning was exactly the wrong thing to do. Press Secretary C.J. Craig is being prepared for a deposition, and her lawyer asks, “Do you know what time it is?” When Craig responds by telling him the time, the lawyer reminds her that he only asked whether she knew what time it was and says “Don't do that. Don't answer questions not asked of you.” Again. Context is everything.

When I first read about Theory of Mind,^4^ I was struck by how much it sounded like the concept of projection, the tendency to mistakenly assume that other people see the world the way you do. Yet, Theory of Mind deficits were being presented as unique to autistic people. In a Theory of Mind experiment, a subject is asked to observe two characters: Sally and Ann. Sally places a marble in a box and leaves the room. Ann moves the marble to another box while Sally is absent. The question the subject must answer: where will Sally look for her marble when she returns? Nonautistic subjects tend to answer correctly that Sally will look for the marble in the box she left it in. Autistic subjects, however, make the mistake of answering that Sally will look for the marble where Ann moved it.

The Sally/Ann test made me think of a game I once played with my mother when I was a teenager in which she would take a comic strip and color in one of the characters so that they appeared to be Black. Her point? Regardless of the intent of the original narrative, it always changed when race entered the picture. What would happen, I wondered, if elements were added that imbued Sally and Ann with some real-world characteristics? What if each was given a backstory, a personality, and a relationship to the other one? Do Sally and Ann know each other, or are they strangers? Do they come from different ethnic or socioeconomic backgrounds? Maybe Sally and Ann are rivals, and “the marble” is a thing of value like money or an important document that is the object of said rivalry.

Admittedly, I am not sure exactly how to design a Sally/Ann test with real-world variables, but I imagine that such an experiment could reveal much about how assumptions, biases, and emotions can, at times, eclipse even the most intact Theory of Mind. Indeed, the Double Empathy Problem^5^ has emerged as a corrective to the concept that the nonautistic Theory of Mind is essentially flawless. According to a recent article in Autism Spectrum News,^6^

The basis of the theory is that a mismatch between two people can lead to faulty communication. This disconnect can occur at many levels, from conversation styles to how people see the world. The greater the disconnect, the more difficulty the two people will have interacting.

In other words, perspective-taking is a two-way street, and nonautistics have just as much difficulty assessing the mental states of autistics as the other way around.

One of the ironies of being both Black and autistic is that the former might have mitigated any tendencies toward literal thinking or problems with perspective-taking I might have had because of the latter. From a young age, I was taught that racism is not always obvious. It is often subtle and can only be detected by reading in-between the lines. In addition, being Black has forced me to develop a double consciousness, a dual awareness of how I view myself versus how I am perceived by the dominant White culture. As writer, scholar, and civil rights leader, W.E.B. Du Bois notes in *The Souls of Black Folk*^7^:

It is a peculiar sensation, this double-consciousness, this sense of always looking at one's self through the eyes of others, of measuring one's soul by the tape of a world that looks on in amused contempt and pity. One ever feels his twoness—an American, a Negro: two souls, two thoughts, two unreconciled strivings; two waring ideals in one dark body, whose dogged strength alone keeps it from being torn asunder (5).

I was once asked whether being Black and autistic meant that I had a triple consciousness. After giving it some thought, I decided no. It is not a question of arithmetic. It does not matter how many characteristics you have that place you outside the mainstream. A double consciousness is what you get whenever you are forced to see things from an alternative perspective. I have long detected the manifestations of a double consciousness in my nondisabled friends as they navigate a world that was not designed for their disabled children. And, during the national racial reckoning that occurred after the murder of George Floyd, I observed the emergence of a double consciousness in my White friends as they empathized with people of color.

Like with Theory of Mind, a double consciousness requires juggling more than one point of view. It can be challenging and, at times, feel like too much of a mental burden to bear. But it can also be a source of motivation and strength. As American author F. Scott Fitzgerald wrote in an essay for Esquire Magazine in 1936^8^:

the test of a first-rate intelligence is the ability to hold two opposed ideas in the mind at the same time, and still retain the ability to function. One should, for example, be able to see that things are hopeless and yet be determined to make them otherwise (41).

My hope moving forward is that researchers adopt a more nuanced view of how autism fits into the boarder human condition. As it stands now, autism research tends to divide the world into two distinct groups—autistics and neurotypicals—and treat them as if they have little in common; this approach only serves to further marginalize autistics by positioning them as an “Other” that no “normal” person could ever really fathom.

But, in truth, autistics and nonautistics share many of the same behaviors and thought patterns. I am not arguing that everyone is a little bit autistic; I am suggesting that the designation of certain characteristics as belonging only to autistics can be dehumanizing. Indeed, research that explored the universality of “uniquely” autistic traits would go a long way toward removing the stigma from autism. It could also yield some fascinating insights about human nature.

## Notes

Gyasi Burks-Abbott is a writer, public speaker, and autism self-advocate. In addition to serving on the boards, committees, and commissions of several autism and disability organizations, Gyasi is a faculty member at Boston Children's Hospital LEND Program that stands for Leadership Education in Neurodevelopmental and Related Disabilities. He graduated from Macalester College in St. Paul, MN, with a BA in English and psychology, and he has an MS in library and information science from Simmons University in Boston. Recently, Gyasi published a book about his life called *My Mother's Apprentice: An Autistic's Rites of Passage*. He lives in Bedford, MA.
